# Induction of Neuron-Specific Degradation of Coenzyme A Models Pantothenate Kinase-Associated Neurodegeneration by Reducing Motor Coordination in Mice

**DOI:** 10.1371/journal.pone.0130013

**Published:** 2015-06-08

**Authors:** Stephanie A. Shumar, Paolo Fagone, Adolfo Alfonso-Pecchio, John T. Gray, Jerold E. Rehg, Suzanne Jackowski, Roberta Leonardi

**Affiliations:** 1 Department of Biochemistry, School of Medicine, West Virginia University, Morgantown, West Virginia, United States of America; 2 Department of Hematology, St. Jude Children’s Research Hospital, Memphis, Tennessee, United States of America; 3 Department of Infectious Diseases, St. Jude Children’s Research Hospital, Memphis, Tennessee, United States of America; 4 Department of Pathology, St. Jude Children’s Research Hospital, Memphis, Tennessee, United States of America; Hertie Institute for Clinical Brain Research and German Center for Neurodegenerative Diseases, GERMANY

## Abstract

**Background:**

Pantothenate kinase-associated neurodegeneration, PKAN, is an inherited disorder characterized by progressive impairment in motor coordination and caused by mutations in *PANK2*, a human gene that encodes one of four pantothenate kinase (PanK) isoforms. PanK initiates the synthesis of coenzyme A (CoA), an essential cofactor that plays a key role in energy metabolism and lipid synthesis. Most of the mutations in *PANK2* reduce or abolish the activity of the enzyme. This evidence has led to the hypothesis that lower CoA might be the underlying cause of the neurodegeneration in PKAN patients; however, no mouse model of the disease is currently available to investigate the connection between neuronal CoA levels and neurodegeneration. Indeed, genetic and/or dietary manipulations aimed at reducing whole-body CoA synthesis have not produced a desirable PKAN model, and this has greatly hindered the discovery of a treatment for the disease.

**Objective, Methods, Results and Conclusions:**

Cellular CoA levels are tightly regulated by a balance between synthesis and degradation. CoA degradation is catalyzed by two peroxisomal nudix hydrolases, Nudt7 and Nudt19. In this study we sought to reduce neuronal CoA in mice through the alternative approach of increasing Nudt7-mediated CoA degradation. This was achieved by combining the use of an adeno-associated virus-based expression system with the synapsin (Syn) promoter. We show that mice with neuronal overexpression of a cytosolic version of Nudt7 (scAAV9-Syn-Nudt7cyt) exhibit a significant decrease in brain CoA levels in conjunction with a reduction in motor coordination. These results strongly support the existence of a link between CoA levels and neuronal function and show that scAAV9-Syn-Nudt7cyt mice can be used to model PKAN.

## Introduction

Syndromes of neurodegeneration with brain iron accumulation (NBIAs) are rare, inherited and genetically defined disorders characterized by an accumulation of iron in the brain and progressive impairment in movement, cognition and vision [1]. Pantothenate kinase-associated neurodegeneration (PKAN) is the most common syndrome among the NBIA disorders, shows an autosomal recessive inheritance and it is caused by mutations in *PANK2* [2], a gene that encodes one of four pantothenate kinase (PanK) isoforms. PanK initiates the synthesis of coenzyme A (CoA), a universal biological cofactor that activates cellular organic acids as acyl-CoA thioesters for their participation in hundreds of metabolic reactions and protein acetylation [3, 4]. The four mammalian PanKs, PanK1α, 1β, 2 and 3 have distinct regulatory properties, tissue distributions and subcellular localizations [3, 5]. Unlike the murine and other mammalian PanK2 homologs, human PANK2 possesses a full length mitochondrial localization signal and localizes to the mitochondria and the nucleus [5, 6]. This enzyme is a major PanK isoform in human brain, and accordingly PKAN symptoms are predominantly limited to the central nervous system with only modest metabolic alterations reported for some PKAN patients [7, 8]. Most of the disease-causing mutations in *PANK2* significantly reduce or abolish the activity of the enzyme [9, 10]. This evidence, combined with the established importance of CoA for mitochondrial bioenergetics and cellular metabolism, suggests that a reduction in CoA and the consequent impairment in neuronal function might be the underlying causes of the neurodegeneration in PKAN patients. The recent discovery of NBIA patients with mutations in *COASY* [11], a downstream gene in the pathway required for CoA synthesis, further supports the connection between reduced neuronal CoA levels and neurodegeneration.

Although *PANK2* mutations were identified as the genetic cause for PKAN in 2001 [2], generating a mouse model for the disease by manipulating PanK activity in mice has proven to be challenging. Inactivation of the homologous *Pank2* gene in mice alters mitochondrial respiration [12] and results in lower brain CoA in the early postnatal period [13]. However, CoA levels normalize in adult animals and the *Pank2* ^*-/-*^ mice do not exhibit neurodegeneration [13, 14]. Global deletion of *Pank1* in addition to *Pank2* results in reduced brain CoA in pups and transient hind limb dragging prior to death at about two weeks of age [13]. *Pank2* knockout mice on a pantothenate deficient diet [15] or a low carbohydrate/high fat diet [16] exhibited movement disorders; however, it is not known whether brain CoA was altered or whether diet-induced perturbations in whole-body glucose homeostasis contributed to the neuromuscular phenotype of the animals [17–20].

Cellular CoA levels are tightly regulated. Feedback regulation of the PanK isoforms by acyl-CoAs controls the biosynthetic pathway [21, 22]. The activity of two nudix hydrolases, Nudt7 and Nudt19, controls CoA degradation into acyl-phosphopantetheine and 3’,5’-ADP [23, 24]. These enzymes reside in the peroxisomes and are abundant in mouse liver and kidney, two organs that, unlike the brain, exhibit high rates of CoA turnover [25]. The objective of this study was to test the hypothesis that lowering CoA levels in neurons could cause symptoms similar to those observed in PKAN patients, independent of dietary manipulations. To achieve a neuron-specific reduction in CoA levels we exploited an adeno-associated virus (AAV)-based expression system and the CoA-degrading activity of Nudt7. Our results demonstrate the existence of a connection between reduced brain CoA and motor coordination in mice fed standard chow. These data support the use of mice overexpressing Nudt7 in neurons as a viable model for PKAN.

## Materials and Methods

### Materials

The cDNA clone (ID 5102473) carrying the full-length sequence of mouse *Nudt7* was purchased from GE Healthcare; pmCHERRY-C1 vector from Clontech; the HEK293 cell line was from the American Type Culture Collection; pET-28a(+) from EMD Millipore; oligonucleotides, pCR2.1 vector, RNA*later*, Pluronic F-68, Lipofectamine 2000, cell culture reagents and citrate buffer pH 6 were from Life Technologies; restriction enzymes were from New England BioLabs. Lipid standards were purchased from Sigma-Aldrich or Avanti Polar Lipids and high performance thin layer chromatography (HPTLC) silica gel 60 plates from EMD Millipore. All other reagents were purchased from Sigma-Aldrich or Fisher Scientific, unless otherwise stated.

### Plasmid Construction

Plasmid scAAV-Syn-GFP was obtained by substituting the CMV promoter of pscAAV-GFP plasmid (Addgene plasmid #32396) [26] with the synapsin-1 promoter [27]. To express the full-length mouse Nudt7 and a truncated form lacking the peroxisomal localization signal, Nudt7cyt, *Nudt7* was amplified from a cDNA clone (ID 5102473) using a common forward primer introducing a 5’ *NdeI* restriction site (5’-CATATGTCGCGACCTTGTGGACTCC-3’) and individual reverse primers introducing a 3’ *NotI* restriction site (5’-GCGGCCGCTCACAACTTGCTTAAAGAATATCTCCAAAGGAAGGTCCTTTCAC-3’ for *Nudt7* and 5’-GCGGCCGCTCATAAAGAATATCTCCAAAGGAAGGTCC-3’ for *Nudt7cyt*). The resultant *Nudt7* and *Nudt7cyt* PCR products were ligated into pCR2.1, excised with *NdeI* and *NotI* and subcloned into pET-28a(+) to generate pKM204 and pKM218, respectively. For the construction of AAV vectors, *Nudt7* and *Nudt7cyt* were amplified with an *EcoRI* restriction site, a Kozak sequence and a FLAG tag at the 5’ end. This long sequence was introduced in two steps using overlapping forward primers (5’-ATGACGACGATAAGAGCAGCGGCATGTCGCGACCTTGTGGACTCCCGGAGCCTGTC-3’) and (5’-GAATTCCCACCATGGATTACAAGGATGACGACGATAAGAGCAGCGGCATGTCGCGACC-3’). The same reverse primers used to generate pKM204 and pKM218 were used to introduce a 3’ *NotI* restriction site. The final PCR products were ligated into pCR2.1 and subcloned into scAAV-Syn-GFP between *EcoRI* and *NotI* sites. For localization studies, *Nudt7* was excised from the cDNA clone above using *PstI* and *ApaI* and ligated into similarly restricted pmCHERRY-C1 vector. The resultant construct was named pAA220. *Nudt7cyt* was amplified using a forward primer introducing a *BglII* restriction site (5’-AGATCTATGTCGCGACCTTGTGGACTCC-3’) and a reverse primer introducing a *BamHI* restriction site (5’-GGATCCTCATAAAGAATATCTCCAAAGG-3’). The PCR product was ligated into pCR2.1 and subcloned into the pmCHERRY-C1 vector within *BglII/EcoRI* sites to produce pKM209.

### Nudt7 and Nudt7cyt Localization, Expression and Enzymatic Activity

The subcellular localization of Nudt7 and Nudt7cyt was determined by confocal live cell imaging after co-transfection of HEK293 cells with the peroxisomal marker pAA344 and either pAA220 or pKM209 as previously described [5]. Recombinant Nudt7 and Nudt7cyt were expressed with an N-terminal hexahistidine tag from pKM204 and pMK218 in BL21(DE3) cells grown for 18–20 h at 16°C following induction with 1 mM isopropyl β-D-1-thiogalactopyranoside. The proteins were purified using standard non-denaturing nickel-nitrilotriacetic acid column chromatography, followed by dialysis in 20 mM Tris-HCl pH 7.5, 300 mM NaCl, 1 mM dithiothreitol overnight. Glycerol was added to the purified proteins to a final concentration of 50% for storage at -20°C. The activity of purified Nudt7 and Nudt7cyt against acetyl-CoA was measured in duplicate as described by Reilly, S-J et al. in a total volume of 40 μl [28]. The amount of 3’,5’-ADP formed was determined by high pressure liquid chromatography (HPLC) on a 4.6 x 150 mm, 3 μm C-18 column (Acclaim 120) kept at 35°C. Elution was performed at 0.7 ml/min as follows: 0–2.5 min, 100% buffer A (50 mM KH_2_PO_4_, 2% acetonitrile); 2.5–9.0 min linear gradient to 100% B (25 mM KH_2_PO_4_, 50% acetonitrile); 9.0–10.0 min isocratic at 100% B; 10.0–15.0 min linear gradient from 100% B to 100% C (3.4 mM KH_2_PO_4_, 80% acetonitrile); 15.0–19.0 min isocratic at 100% C; 19.0–32.0 min return to 100% A and column equilibration. The formation of 3’,5’-ADP was monitored by measuring the absorbance at 260 nm and quantified using a curve of 3’,5’-ADP (Sigma-Aldrich) standards processed as the reaction mixtures. To test the Nudt activity in brain homogenates, ∼100 mg of tissue were homogenized in 20 mM Tris-HCl pH 8.0, centrifuged at 1500xg for 5 min and the supernatant incubated in reaction mixtures containing 100 mM Tris-HCl pH 8.0, 5 mM MgCl_2_, 195 μM [acetyl-1-^14^C]-acetyl-CoA (PerkinElmer, specific activity 2.1 mCi/mmol) and 32–250 μg of protein in a total volume of 40 μl. The reaction mixtures were incubated at 37°C for 30 min, stopped by the addition of 4 μl of 250 mM EDTA and analyzed by thin layer chromatography as previously described [29].

### AAV Production

Plasmids pscAAV-GFP, scAAV-Syn-GFP, scAAV-Syn-Nudt7cyt were used to produce AAV serotype 9 particles in HEK293T cells as previously described [30]. The viral particles were purified using an iodixanol gradient [31] followed by ion exchange chromatography. Briefly, the virus-containing fractions from the iodixanol gradient were collected, diluted with 20 mM sodium acetate buffer, pH 5.5 and loaded onto a 10 ml POROS HS 50 column (Life Technologies). The virus was eluted with a linear gradient of NaCl from 0.010 to 1.0 M in 20 mM acetate buffer, pH 5.5 and collected in tubes containing 0.05 volumes of 400 mM Bis-Tris propane, pH 9.0. The buffer was exchanged to phosphate-buffered saline (PBS) and Pluronic-F68 added to a final concentration of 0.02% before quantifying the virus as previously described [32].

### Ethical Statement

All animal procedures described in this study were performed according to protocol 323 and specifically approved by the St. Jude Children’s Research Hospital Institutional Animal Care and Use Committee.

### Animal Studies

Mice were fed standard chow (LabDiet 5013) and maintained at 22 ± 2°C with a humidity of 50% ± 10% and a 14 h light/10 h dark cycle. Pregnant C57BL/6 females at gestational day 17 were obtained from Charles River, housed in individual cages and closely monitored until delivery. Between 18 and 24h after birth, pups of either sex were randomly assigned to the Nudt7cyt or GFP group and injected through the superficial temporal vein with 3.0*10^11^ genome copies of the respective scAAV9 in PBS (100 μl) containing 0.001% pluronic F-68 [33]. The grip strength of the front limbs was measured using a grip-strength meter (Ugo Basile, model 47200). The rotarod test was conducted using an accelerating rotarod (Ugo Basile, model 47600). The mice were trained over 2 consecutive days by placing them on the drum rotating at 4, 8, 15, 24, 30 and 40 rpm for 60 sec, allowing 5 min rest before each increase in speed. During the following 3 days the mice were tested for a maximum of 300 seconds at each speed, with 5 min breaks in between. For each mouse, the time spent on the rotarod before falling at a particular speed was recorded and averaged over the 3 days. For all the experiments, the number of mice/group is indicated in the correspondent figure or figure legend.

### CoA Analysis, Western Blotting and RT-PCR

Tissue was collected from anesthetized mice and either immediately frozen in liquid nitrogen or preserved in RNA*later*. Frozen tissue was stored at -80°C until used. After removal of the olfactory bulb, the brain was consistently sectioned into 2 parts per hemisphere using a coronal brain mold. Matching brain sections from different mice were used for all the analyses. For CoA analysis, a portion of the brain from 5 week-old (1 mouse/group), 9 week-old (3 mice/group) and 17 week-old (1 mouse/group) mice was homogenized in 1 mM potassium hydroxide (2 ml). The pH was further adjusted to ∼12 and the homogenates were heated at 55°C for 2 h to hydrolyze all acyl-CoAs to free CoA. After this incubation, the pH of the samples was decreased to around 8 with 1 M Trizma-HCl and monobromobimane (1 μmole) added to convert free CoA into a fluorescent derivative [34]. The samples were incubated in the dark at room temperature for 2 h, acidified with glacial acetic acid, and partially purified through a 2-(2-pyridyl)ethyl-functionalized silica gel column (Supelco) [35]. After drying under a nitrogen flow, the samples were re-suspended in water and analyzed by HPLC using a Waters Alliance e2695 system equipped with a 2489 UV/Vis detector and a Gemini 4.6 x 150 mm, 3 μm C-18 column (Phenomenex) kept at 35°C. Elution was performed as follows: 0–2.0 min, 90% buffer A (50 mM KH_2_PO_4_, pH 4.6), 10% buffer B (acetonitrile); 2.0–9.0 min concave gradient to 25% B; 9.0–23 min linear gradient to 40% B; 23.0–30 min liner gradient to 90% A and column equilibration in 90% A. Monobromobimane-CoA was detected by monitoring the absorbance at 393 nm and quantified using a standard curve. For RT-PCR, a portion of the brain was processed and analyzed as previously described [13]. The previously published primers for Nudt7 were used to estimate the levels of Nudt7cyt mRNA [20]. Primers for peroxisome proliferative activated receptor, gamma, coactivator 1 alpha (*Pgc1α*), phosphoglycerate kinase 1 (*Pgk1*) and acyl-CoA synthetase long-chain family member 1 (*Acsl1*) were previously published [13, 36]. Other primer sequences used were: superoxide dismutase 1 (*Sod1*), forward 5’-CAAGCGGTGAACCAGTTGTG-3’ and reverse 5’-TGAGGTCCTGCACTGGTAC-3’; superoxide dismutase 2 (*Sod2*), forward 5’-GCCTGCACTGAAGTTCAATG-3’ and reverse 5’-ATCTGTAAGCGACCTTGCTC-3’; glucuronidase β (*GusB*), forward 5’-ATAAGACGCATCAGAAGCCG-3’ and reverse 5’-ACTCCTCACTGAACATGCGA-3’; lactate dehydrogenase 1 (*Ldh1*), forward 5’-CTTGACCTACGTGGCTTGGAA-3’ and reverse 5’-GTGCCCAGTTCTGGGTTAAGA-3’. For western blot analysis, tissues were homogenized in cold RIPA buffer and centrifuged at 10,000xg for 10 min at 4°C. The Nudt7 antibody was generated and used as previously reported [37]. The HRP-conjugated GFP antibody (Santa Cruz Biotechnology) were used at 1:1000 dilution; the glyceraldehyde-3-phosphate dehydrogenase (GAPDH) antibody (Abcam) at 1:1500 dilution. Bound primary antibodies were detected by chemiluminescence with HRP-conjugated goat anti-rabbit IgG at 1:5000 dilution (GE Healthcare).

### Lipid Analysis

Lipids were extracted from brain samples (30–40 mg) as previously described [38] and fractionated using HPTLC silica plates and the solvent system indicated below. Resolved lipid bands were visualized by spraying with primulin dye followed by fluorescence detection using a Typhoon 9410 (GE Healthcare) imaging system [39]. The mass of each major lipid class was determined by comparing the band intensities from experimental samples with 2-fold serial dilutions of lipid standards loaded on the same TLC plate. Phosphatidylethanolamine, phosphatidylcholine and sphingomyelin were separated using chloroform:methanol:acetic acid:water (30:20:2:1; v:v:v:v); cholesterol was resolved using chloroform:methanol:acetic acid (98:2:1; v:v:v).

### Immunohistochemistry

Representative tissue samples were collected and fixed in 10% neutral buffer formalin, embedded in paraffin wax and sectioned at 4 μm. Unstained tissue sections were collected on positive charged slides and deparaffinized at 60°C for 30 min. Antigen retrieval was performed in citrate buffer pH 6 at 110°C for 15 min. The slides were sequentially incubated for 5 min with 5% hydrogen peroxide, 30 min with Background Sniper (BioCare Medical), 1 h with the GFP antibody at 1:200 dilution (Life technologies), 30 min with Rabbit Envision (DAKO), and 10 min with 3,3'-diaminobenzidine (DAKO). The slides were counterstained with Meyer’s Hematoxylin for 3 min at 1:10 dilution (ThermoShandon) and dehydrated. All assays were done on an Autostainer 720 (ThermoShandon) at room temperature.

### Statistical Analysis

Unless otherwise stated, all data are presented as the mean with ± the standard error. Statistical significance was calculated by unpaired two-tailed Student's t test using GraphPad Prism 6 (GraphPad Software).

## Results and Discussion

### Removal of the Peroxisomal Targeting Signal Alters Nudt7 Localization but not its Activity

**Fig 1:**
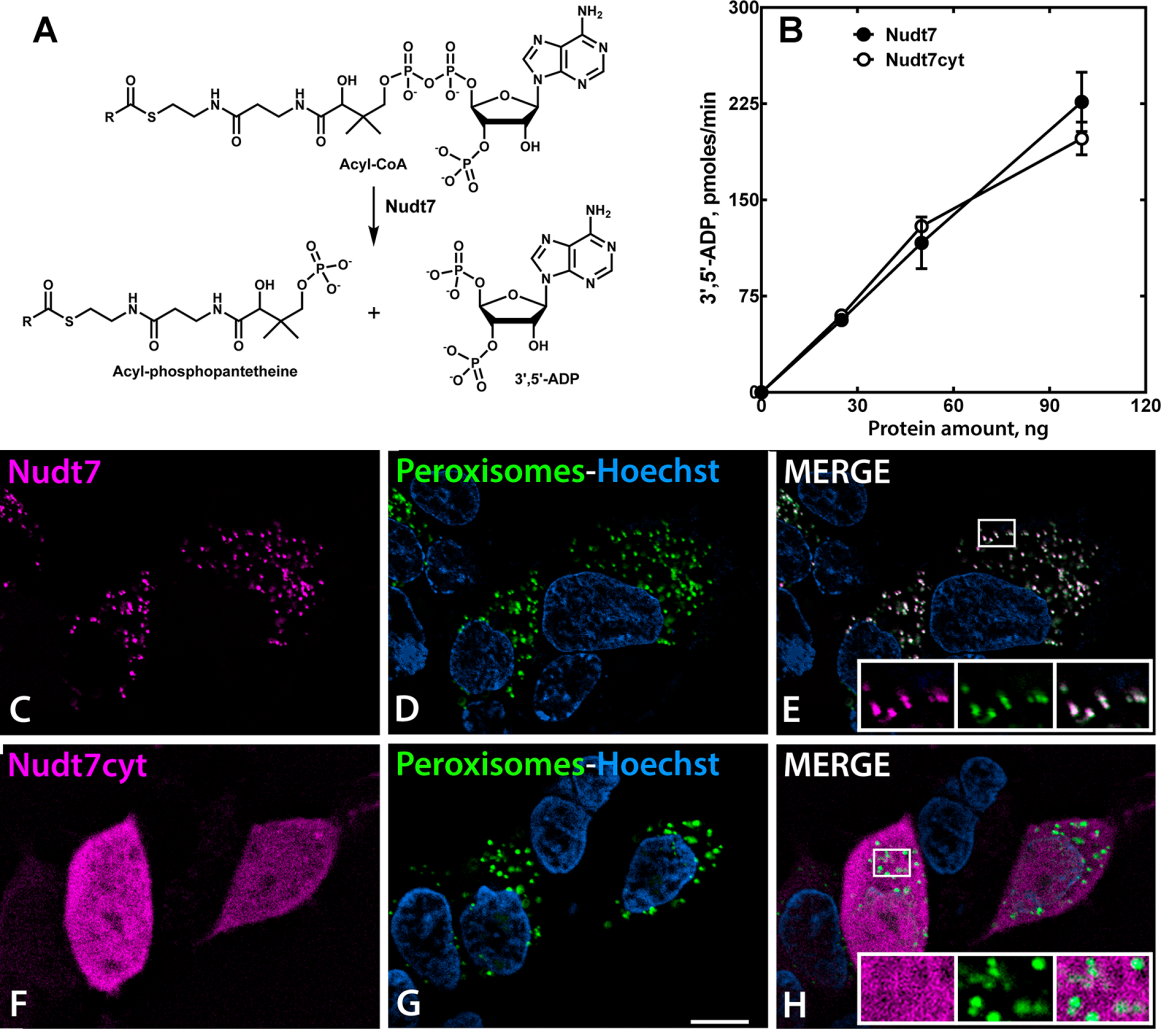
Nudt7 and Nudt7cyt activity and localization. (A) Nudt7 hydrolyzes the phosphodiester bond in acyl-CoA species producing acyl-phosphopantetheine and 3’,5’-ADP. (B) Enzymatic activities of purified mouse Nudt7 (closed circles) and Nudt7cyt (open circles). Data are reported as the mean ± the range. (C-H) HEK293 cells were transfected with expression plasmids encoding mCherry fused to the N-terminus of Nudt7 (C-E) or Nudt7cyt (F-H) as described in ‘Material and Methods’. mCherry-Nudt7 and mCherry-Nudt7cyt proteins are shown in magenta (C, F) and cell nuclei are stained with Hoechst 33342 in blue (D, E, G, H). Co-transfection with a plasmid encoding green fluorescent protein (GFP) with a C-terminal SKL (GFP-SKL) (D, G) designates the peroxisomes shown in green. GFP-SKL colocalizes with Nudt7 (E, shown in white) but not with Nudt7cyt (H). The mCherry-Nudt7, mCherry-Nudt7cyt and GFP-SKL proteins were visualized by live-cell confocal fluorescent microscopy. Scale bar, 10 μm.

Cellular CoA is present in three distinct pools in the cytosol, mitochondria and peroxisomes [40, 41]. CoA synthesis is completed in the cytosol [42] and only ∼10% of the cytosolic CoA is transported into mitochondria and peroxisomes in liver (S. Jackowski, unpublished observation). Nudt7 catalyzes the Mg^2+^-dependent cleavage of acyl-CoA species into 3’,5’-ADP and the corresponding acyl-phosphopantetheine moieties (**Fig 1A**). The enzyme is highly specific for CoA species and has a C-terminal peroxisomal targeting signal type 1 (PST1), the tripeptide SKL, that directs the protein to the peroxisomes and restricts its CoA-degrading activity to this subcellular compartment [24] (**Fig 1C, 1D and 1E**). Since degradation of the cytosolic CoA pool could also potentially affect the peroxisomal and mitochondrial pools, we generated a cytosolic Nudt7 mutant, Nudt7cyt, lacking the PST1. Expression and purification of Nudt7cyt resulted in a soluble protein with activity similar to the wild-type Nudt7 (**Fig 1B**). Localization of Nudt7cyt in HEK293 cells using confocal microscopy confirmed its exclusion from the peroxisomes and revealed a diffused cytosolic localization (**Fig 1F, 1G and 1H**).

### Neuron-specific Expression of Nudt7cyt Results in Reduced Brain CoA and Motor Function

**Fig 2:**
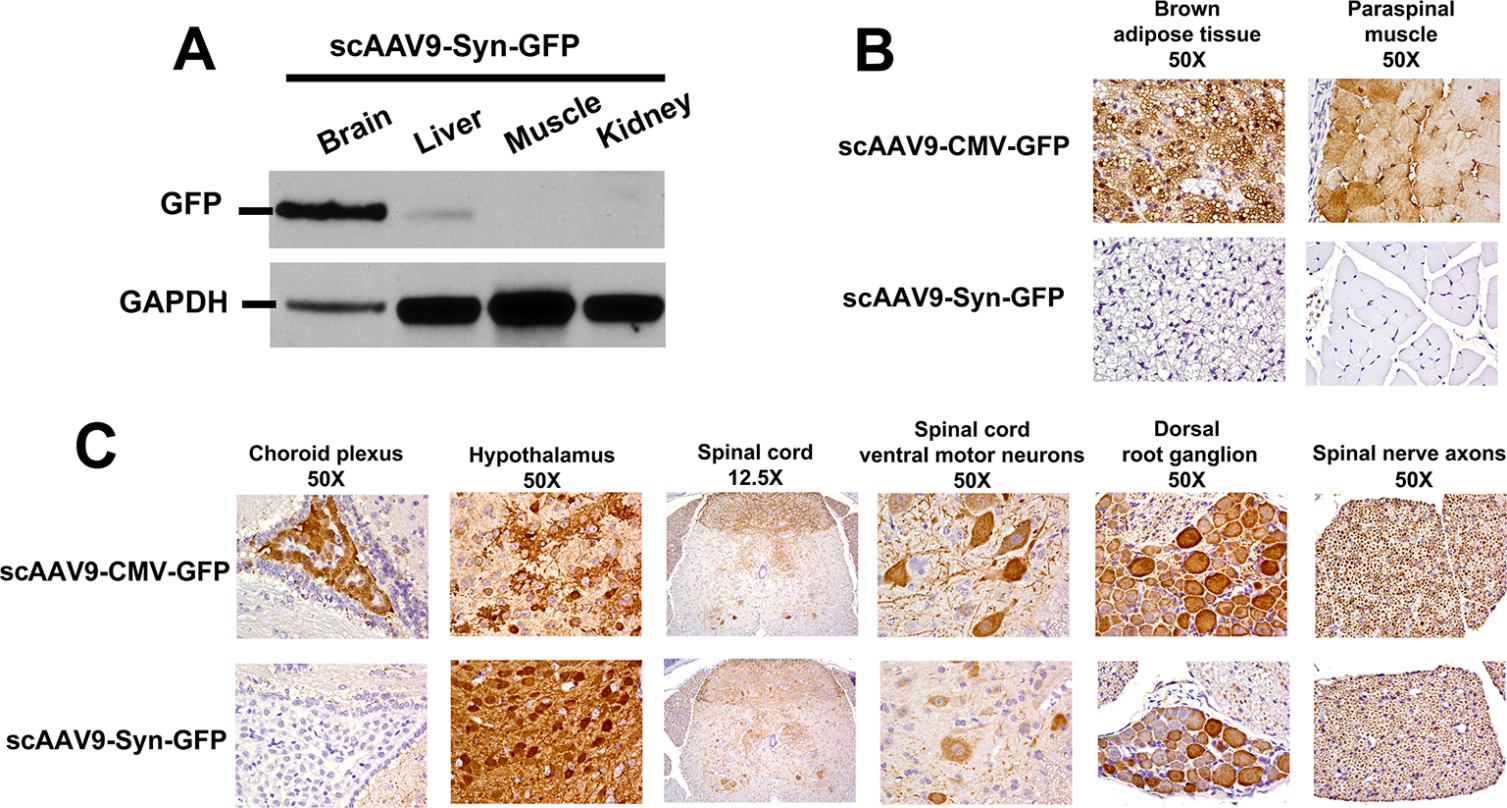
GFP expression driven by the CMV or Syn promoter. (A) Western blotting analysis of GFP in tissues from animals injected with AAV9-Syn-GFP. GAPDH was used as the loading control. Immunohistochemistry of (B) brown adipose tissue and paraspinal muscle and (C) central nervous system regions. GFP expression (brown) was driven by the CMV or the Syn promoter. AAV particles were injected as described in ‘Material and Methods’ and mice were analyzed 4 weeks thereafter. The results are representative of 2 or more animals. The magnifications are indicated.

Intravenous injection of postnatal day 1 (P1) pups with AAV serotype 9 (AAV9) was recently shown to achieve high transduction of motor neurons [33, 43]. Injection of a self-complementary (sc) AAV9 carrying green fluorescent protein (GFP) with expression driven by the cytomegalovirus (CMV) promoter (scAAV9-CMV-GFP) resulted in robust GFP expression in brown adipose tissue, muscle, brain and spinal cord (**Fig 2B and 2C**). Replacement of the CMV promoter with the synapsin-1 promoter [27] (scAAV9-Syn-GFP) showed an expression pattern consistent with neuron-specific GFP expression (**Fig 2A, 2B and 2C**), in agreement with reports by other groups [44, 45]. In order to dissect the role of CoA in neuronal function, we assembled the scAAV-Syn-Nudt7cyt, scAAV-Syn-Nudt7 and scAAV-Syn-GFP (control) vectors and injected multiple cohorts of mice with the derived scAAV9 particles. In pilot experiments using the rotarod test to assess motor coordination and balance, mice injected with scAAV9-Syn-Nudt7cyt exhibited a similar but more robust phenotype compared to mice overexpressing wild-type Nudt7 (**S1 Fig**); thus, all subsequent experiments were conducted using the scAAV9-Syn-Nudt7cyt virus.

**Fig 3:**
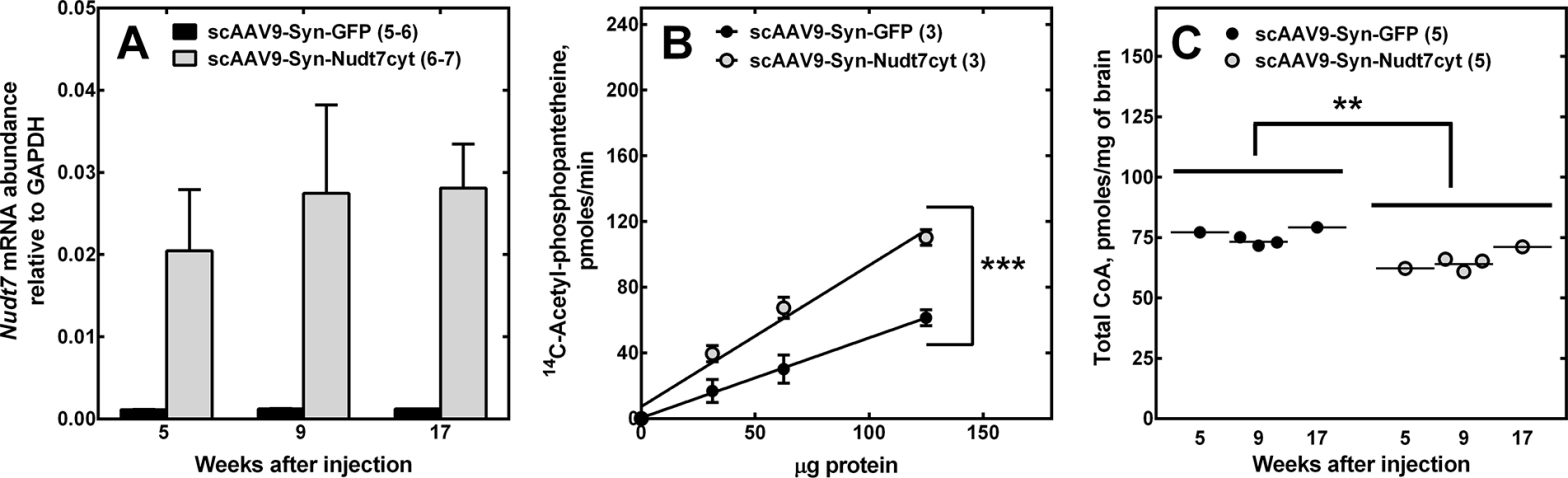
Nudt7cyt and CoA levels in brains of animals injected with scAAV9-Syn-Nudt7cyt. (A) Nudt7 mRNA levels in mice injected with scAAV9-Syn-Nudt7cyt or scAAV9-Syn-GFP as normalized to GAPDH. AAV particles were injected as described in ‘Materials and Methods.’ (B) Overexpression of Nudt7cyt was confirmed by western blot analysis (inset) and measurement of CoA-degrading activity in brain homogenates. (C) Brain CoA was measured in 5–17 week-old mice, as indicated, injected with scAAV9-Syn-Nudt7cyt or AAV9-Syn-GFP as described in ‘Material and Methods’. Numbers in parenthesis indicate the total number of animals used for the measurements. Data are reported as the mean ± the standard error. *, *p*<0.05; **, *p*<0.01; ***, *p*<0.001.

**Fig 4:**
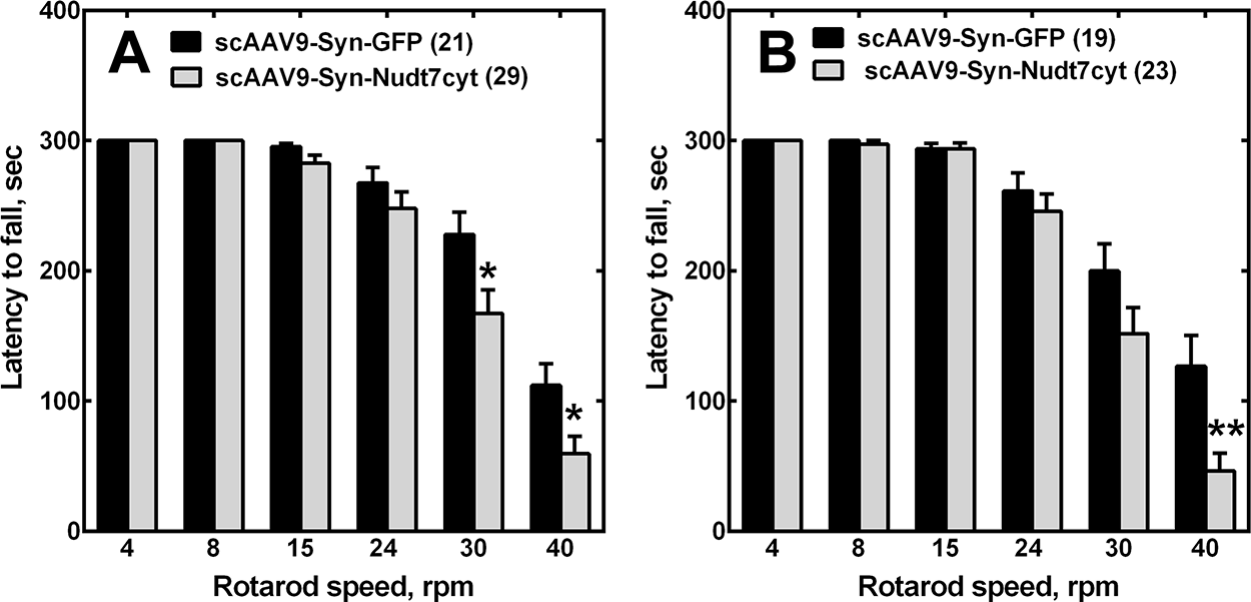
Reduced motor coordination in mice with neuron-specific overexpression of Nudt7cyt. Mice were analyzed for their performance on the rotarod test at (A) 9 and (B) 17 weeks of age after injection of the AAV9 particles at P1. Numbers in parentheses indicate the number of animals used for the measurement. Data are reported as the mean ± the standard error. *, *p*<0.05; **, *p*<0.01.

The mice injected at P1 with scAAV9-Syn-Nudt7cyt developed normally and did not exhibit any gross motor abnormality compared to the controls. Five weeks after the AAV injections, brain RT-PCR analysis showed a 20-fold increase in *Nudt7* transcripts in the scAAV9-Syn-Nudt7cyt mice compared to the GFP controls, and these high transcript levels were sustained for at least 17 weeks (**Fig 3A**). Nudt7cyt protein (**Fig 3B, inset**) increased in correspondence with the increased transcript levels but the CoA-degrading activity in brain homogenates increased only 2-fold (**Fig 3B**). In light of the weak correlation between Nudt7cyt expression and activity in brain homogenates there is a possibility that localization of Nudt7 in the cytosolic environment and/or the presence of inhibitory components in the unpurified brain homogenates may dampen its activity. However, any mechanisms of regulation of Nudt7 activity are unknown at this time. Regardless, brain homogenates from mice injected with scAAV9-Syn-Nudt7cyt exhibited a significant 15% decrease in total CoA (free plus acyl-CoAs) (**Fig 3C**). This is expected to be an underestimation of the reduction in CoA that occurred in neurons as other cell types that did not express Nudt7cyt contributed to the CoA measurement in brain homogenates. The AAV-treated mice were analyzed for forelimb grip strength at 8 and 16 weeks of age, while the rotarod test was conducted one week after each grip strength test. Mice injected with scAAV9-Syn-Nudt7cyt exhibited a significant reduction in rotarod performance (**Fig 4**) but no reduction in grip strength (data not shown), indicating that muscle weakness was not a major contributor to the reduction in motor coordination. Instead, this phenotype correlated with reduced brain CoA (**Fig 3C**), supporting the existence of a link between reduced neuronal CoA and motor function in mice.

**Fig 5:**
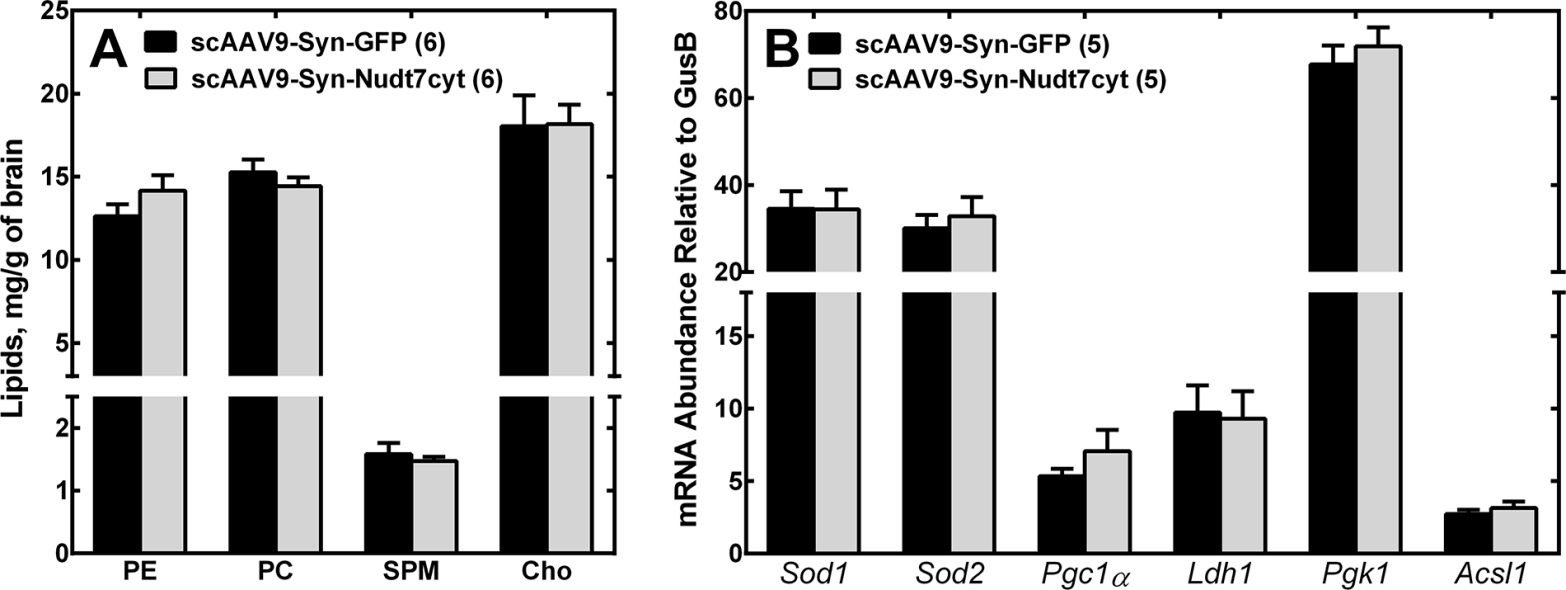
Brain lipid and RT-PCR analyses. Brains from 17 week-old mice were analyzed for (A) lipid composition and (B) transcript levels of selected genes involved in mitochondrial function, metabolism and response to oxidative stress. Abbreviations used are: PE, phosphatidylethanolamine; PC, phosphatidylcholine; SPM, sphingomyelin; Cho, cholesterol. Transcripts were measured for the following genes: *Sod1* and *Sod2*, superoxide dismutase 1 and 2; *Pgc1α*, peroxisome proliferative activated receptor, gamma, coactivator 1 alpha; *Ldh1*, lactate dehydrogenase 1; *Pgk1*, phosphoglycerate kinase 1; *Acsl1*, acyl-CoA synthetase long-chain family member 1. Numbers in parentheses indicate the number of animals used for the measurements. Data are reported as the mean ± the standard error.

Drosophila mutants with global reduction in the expression of CoA biosynthetic enzymes, reduced motor coordination and presumably lower CoA exhibited significantly lower triglyceride and phospholipid levels [46]. We did not find any alteration in the major brain lipid components in mice overexpressing Nudt7cyt (**Fig 5A**). There is not a consensus as to the lipid defects associated with neurodegeneration and data are not available on the brain lipid composition in PKAN patients. Since CoA plays a major role in mitochondrial function and oxidative energy metabolism, we analyzed transcript levels for enzymes involved in antioxidant responses, mitochondrial biogenesis and function, carbohydrate and lipid metabolism (**Fig 5B**). Some of these transcripts were significantly altered in other neurological disorders characterized by brain iron accumulation and mitochondrial dysfunction such as Huntington's and Parkinson’s diseases [47–49]. No difference was detected between scAAV9-Syn-Nudt7cyt mice and controls; however, studies in the liver have shown that reduced CoA levels affect the output of metabolic pathways by limiting substrate availability independent of transcriptional changes [20, 37], and a similar mechanism could be involved in neurons. Further studies will be required to identify the pathways that are perturbed by the decrease in neuronal CoA and that cause the reduction in motor coordination in mice overexpressing Nudt7cyt.

Although several genetic and dietary manipulations have been used to reduce PanK activity or CoA synthesis in mice, the connection between neuronal CoA levels and neurodegeneration has not been conclusively established yet. Indeed, CoA data are not available from studies where special diets were used to induce a neuromuscular impairment in the *Pank2* ^*-/-*^ mice [15, 16]. The major finding of this study was that a reduction in neuronal CoA in the brain correlated with a significant reduction in motor coordination, one of the hallmarks of PKAN. Importantly, no dietary or genetic manipulations that reduced CoA in non-neuronal tissues were used in this model, thereby eliminating the confounding condition of impaired whole-body glucose homeostasis on neuromuscular performance. The phenotype of the scAAV9-Syn-Nudt7cyt mice was relatively mild when compared to that of PKAN patients. It is possible that a portion of the acyl-phosphopantetheines produced by Nudt7cyt may be hydrolyzed to phosphopantetheine by cellular thioesterases and recycled back to CoA by CoA synthase [50]. Additionally, reduction of the CoA levels driven by the overexpression of Nudt7cyt or impaired PANK2 activity would be expected to release the feedback inhibition of the remaining PanKs and lead to a compensatory increase in CoA synthesis [21, 22]. Since PanK is the established rate-limiting step of the pathway, an increase in endogenous PanK activity, but not necessarily PanK protein, is expected to elevate CoA levels without the accumulation of pathway intermediates [22]. Perhaps the capacity of this CoA-buffering system would be significantly more impaired in patients with disease-causing *PANK2* mutations. Alternatively, altered metabolism or functions in other cell types like astrocytes [51], in addition to neurons, could contribute to the neurodegeneration in patients with PKAN disease. There is currently no available treatment for PKAN and, in spite of these caveats, the availability of the scAAV9-Syn-Nudt7cyt mice and a more in depth analysis of their phenotype will allow for the identification of the CoA-dependent molecular mechanisms that underlie the disease and accelerate the development of a cure.

## Supporting Information

S1 Fig Rotarod performance in mice with neuron-specific overexpression of Nudt7 or Nudt7cyt. Mice were analyzed for their performance on the rotarod test (A) 9 and (B) 17 weeks after injection of the AAV9 particles at P1. Mice injected with scAAV9-Syn-Nudt7 or scAAV9-Syn-Nudt7cyt showed a similar reduction in motor coordination at 9 weeks of age; however, mice with neuronal overexpression of Nudt7cyt exhibited a strong trend towards a reduced performance also at 17 weeks of age. Numbers in parentheses indicate the number of animals used for the measurement. Data are reported as the mean ± the standard error. *, p<0.05; **, p<0.01.
(TIF)
