# Supplementary figures and images for: Induction of Neuron-Specific Degradation of Coenzyme A Models Pantothenate Kinase-Associated Neurodegeneration by Reducing Motor Coordination in Mice
Source: PLoS One. 2015 Jun 8;10(6):e0130013. doi: 10.1371/journal.pone.0130013 (PMC4460045; doi:10.1371/journal.pone.0130013)

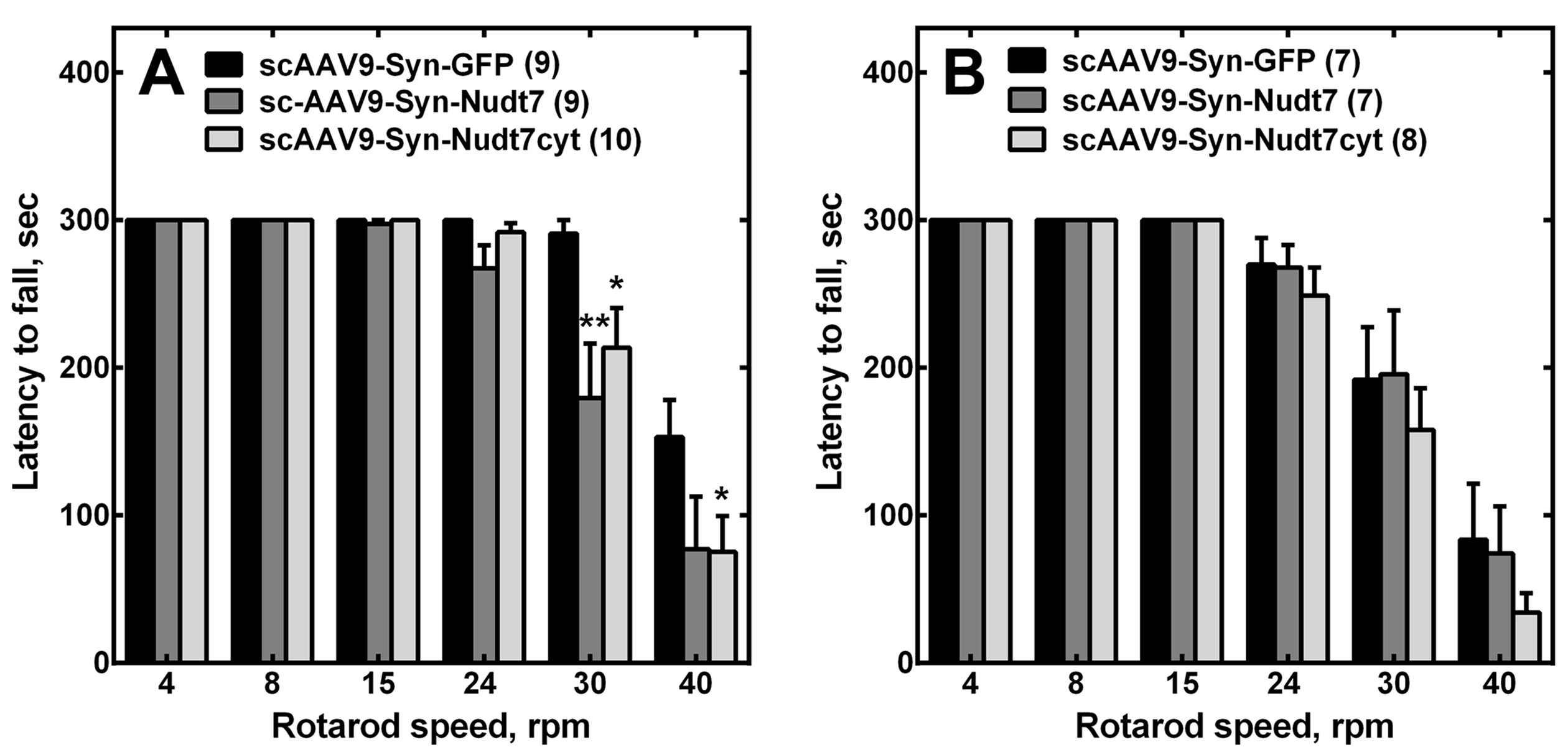

Supplement: S1 Fig — Mice were analyzed for their performance on the rotarod test (A) 9 and (B) 17 weeks after injection of the AAV9 particles at P1. Mice injected with scAAV9-Syn-Nudt7 or scAAV9-Syn-Nudt7cyt showed a similar reduction in motor coordination at 9 weeks of age; however, mice with neuronal overexpression of Nudt7cyt exhibited a strong trend towards a reduced performance also at 17 weeks of age. Numbers in parentheses indicate the number of animals used for the measurement. Data are reported as the mean ± the standard error. *, p<0.05; **, p<0.01. (TIF) [file pone.0130013.s001.tif]
